# PD25 Innovation In Facial Palsy Treatment: The Costs And Benefits Of Telerehabilitation Introduced Into Physical Therapy Pathways

**DOI:** 10.1017/S0266462324002897

**Published:** 2025-01-07

**Authors:** Ala Szczepura, Amir J Khan, Charles Nduka, Catriona Neville, Helen Martin, Karen Johnson, Samuel W Oxford, Shea Palmer, Nikki Holliday, Chris Bark, David Thomson, Hema Mistry

## Abstract

**Introduction:**

The incidence of facial palsy has been rising worldwide, with recent evidence emerging of links to COVID-19 infection. To date, guidance on cost-effective treatments is limited to medication (prednisolone). In terms of physical therapy, neuromuscular retraining (NMR) to restore balanced facial function has been most widely evaluated, but not in terms of cost effectiveness. The added value of telerehabilitation is unknown.

**Methods:**

A multistage technology assessment was conducted, which included the following:

- a national survey of current therapy pathways in the UK and patients’ and clinicians’ views on the benefits and challenges of telerehabilitation;
- a systematic review of clinical effectiveness trials evaluating facial NMR therapy;
- calculation of long-term morbidity costs (national economic burden) based on incidence, patient recovery profiles, health-related quality of life, and national facial palsy treatment costs (valuation of clinical improvements in monetary terms was provided by a national Delphi panel); and
- evaluation of the cost effectiveness of telerehabilitation (remote monitoring wearables) added to current face-to-face NMR delivery.

**Results:**

Nationally, approximately five percent of patients with facial palsy (17% of unresolved cases) are referred for facial NMR. The long-term economic burden associated with unresolved cases is estimated to range from GBP351 (EUR417) to GBP584 (EUR692) million, indicating substantial savings if long-term recovery can be improved. Medical treatment costs are GBP86.34 (EUR102) million per annual cohort, and physical and psychological therapy costs are GBP643,292 (EUR762,561). Economic modeling showed that telerehabilitation was cost effective, producing a health gain and a cost-saving of GBP468 (EUR555) per patient. If scaled to the national level for all patients who do not recover fully, an annual saving of GBP3.075 (EUR3.65) million is possible.

**Conclusions:**

Economic modeling indicates that NMR could improve patient outcomes and reduce costs. The national survey demonstrated that access to NMR therapy services is limited, so introduction of telerehabilitation could improve access for currently underserved populations. Future clinical trials need to incorporate economic evaluations to help inform decision-making.

